# Comparison of survival, acute toxicities, and dose–volume parameters between intensity‐modulated radiotherapy with or without internal target volume delineation method and three‐dimensional conformal radiotherapy in cervical cancer patients: A retrospective and propensity score‐matched analysis

**DOI:** 10.1002/cam4.4439

**Published:** 2021-11-24

**Authors:** Yu‐Qin Liang, Sen‐Quan Feng, Wen‐Jia Xie, Qiong‐Zhi Jiang, Yan‐Fen Yang, Ren Luo, Elizabeth A. Kidd, Tian‐Tian Zhai, Liang‐Xi Xie

**Affiliations:** ^1^ Department of Radiation Oncology Xiang’an Hospital of Xiamen University Xiamen China; ^2^ Department of Radiation Oncology Cancer Hospital of Shantou University Medical College Shantou China; ^3^ Department of Radiation Oncology University of Groningen University Medical Center Groningen Groningen The Netherlands; ^4^ Department of Science and Education Xiang’an Hospital of Xiamen University Xiamen China; ^5^ Department of Radiation Oncology Faculty of Medicine University of Freiburg Freiburg Germany; ^6^ Faculty of Biology University of Freiburg Freiburg Germany; ^7^ Department of Radiation Oncology Stanford University Stanford California USA

**Keywords:** cervical cancer, hematological toxicity, high‐dose rate brachytherapy, intensity‐modulated radiotherapy, internal target volume, propensity score‐matched analysis

## Abstract

**Background:**

To evaluate whether the use of the internal target volume (ITV) delineation method improves the performance of intensity‐modulated radiotherapy (IMRT) and three‐dimensional conformal radiotherapy (3DCRT) in terms of survival, acute toxicities, and dose–volume parameters.

**Methods:**

A total number of 477 cervical cancer patients who received concurrent chemoradiotherapy (CCRT) from January 2012 to December 2016 were retrospectively analyzed. They were divided into four groups: the non‐ITV (N‐ITV) + IMRT, ITV + IMRT, N‐ITV + 3DCRT, and ITV + 3DCRT groups, with 76, 41, 327, and 33 patients, respectively. Survival analysis was performed with the Kaplan–Meier and the log‐rank tests, and acute toxicity analysis was performed with the chi‐squared test and the binary logistic regression test. Using the propensity score matching (PSM) method, 92 patients were matched among the four groups, and their dose–volume parameters were assessed with the Kruskal–Wallis method.

**Results:**

The median follow‐up time was 49 months (1–119) for overall survival (OS). The 5‐year OS rate was 66.4%. The ITV delineation method was an independent prognostic factor for OS (HR [95% CI]: 0.52 [0.27, 0.98], *p* = 0.044) and progression‐free survival (PFS) (HR [95% CI]: 0.59 [0.36, 0.99], *p* = 0.045). The ITV + IMRT group had the lowest incidence rate (22%) and the N‐ITV + IMRT group had the highest incidence rate of grade ≥3 hematological toxicity (HT) (46.1%) among the four groups. The pelvic bone marrow relative V10, V20, and V30 in the N‐ITV + IMRT group was higher than those in the ITV + IMRT and N‐ITV + 3DCRT groups (*p* < 0.05).

**Conclusions:**

The use of ITV for IMRT treatment planning was associated with improved overall survival and progression‐free survival, with lower HT rate.

## Graphical abstract

The use of ITV for IMRT treatment planning was associated with improved overall survival and progression‐free survival, with lesser HT.

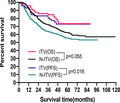

## 1 INTRODUCTION

According to the International Agency for Research on Cancer, cervical cancer has the fourth highest incidence and mortality rates in women worldwide.^1^ There were 569.8 thousand new cervical cancer cases and 311.4 thousand cervical cancer deaths in 2018. Currently, concurrent chemoradiotherapy (CCRT) is the standard treatment for locally advanced cervical cancer.^2^

With the advancement of radiotherapy techniques, IMRT and volumetrically modulated arc therapy (VMAT) are often used for the treatment of cervical cancer. The purpose of precise treatment is to reduce the occurrence of side effects. Many studies^3^, ^4^, ^5^, ^6^, ^7^ have shown that IMRT is associated with higher survival rates and fewer side effects than 3DCRT, including gastrointestinal, genitourinary toxicity, and hematological toxicity. Considering uterine movement^8^, ^9^ and its influences on the bladder and rectum, a consensus on IMRT was published in 2011 for patients receiving definitive CCRT.^10^ Some have proposed creating an ITV to help address the challenge of bladder filling status and vaginal movement, but this has not been supported by all,^9^, ^11^, ^12^, ^13^, ^14^ and there is a lack of data evaluating the disease outcomes and side effects of using an ITV with IMRT.^15^, ^16^ Related studies^11^, ^12^, ^15^, ^17^, ^18^, ^19^, ^20^, ^21^, ^22^ mainly assessed the advantages of ITV from organ motion or contouring margins.

To compare the clinical difference of whether the use of the internal target volume (ITV) delineation method in intensity‐modulated radiotherapy (IMRT) and three‐dimensional conformal radiotherapy (3DCRT) is different in terms of survival, acute toxicities, and dose–volume parameters, this study was conducted by analyzing data from 477 cervical cancer patients treated at a single institution from January 2012 to December 2016.

## 2 METHODS

### 2.1 Patients

**FIGURE 1:**
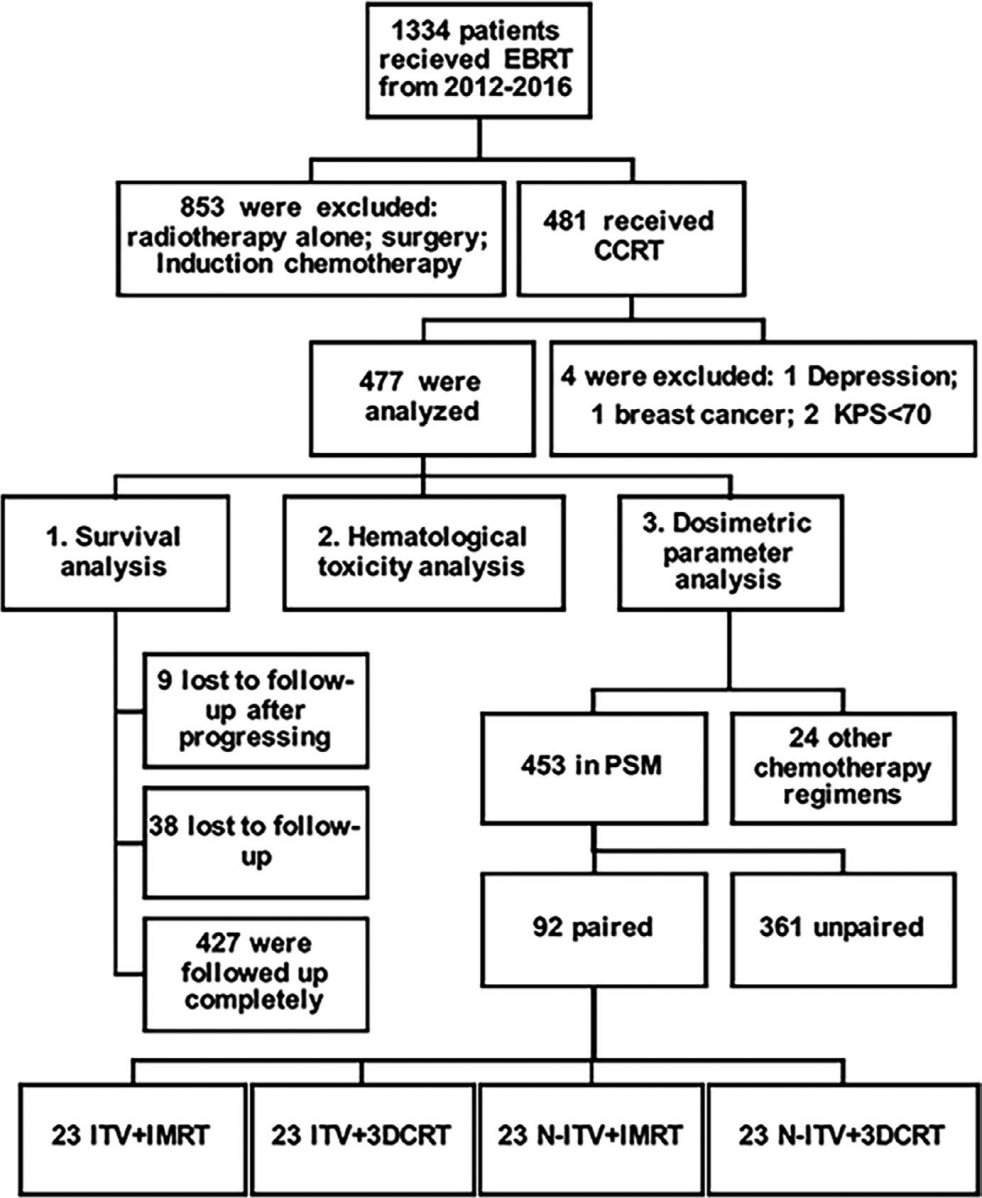
Flow chart of the present study. 3DCRT, three‐dimensional conformal radiotherapy; CCRT, concurrent chemoradiotherapy; EBRT, external beam radiotherapy; IMRT, intensity‐modulated radiotherapy; ITV, internal target volume; KPS, Karnofsky score; N‐ITV, non‐internal target volume; PSM, propensity score matching

The study was a retrospective review from a single institution. A total of 1334 patients without distant metastasis received external beam radiotherapy (EBRT). Figure 1 summarizes the selection of the patients. Among them, 477 patients with 2014 FIGO stage IB2 to IVA and some earlier stage patients who refused surgery were included. All patients receiving definitive CCRT were analyzed. A total of 427 patients had complete follow‐up data, 9 patients ceased contact after disease progression, and 41 patients were lost to follow‐up.

Among all the included patients, the median age was 54 years (range from 26 to 79). In 35 patients, the maximum tumor diameter was unknown due to unclear tumor edges. Pelvic magnetic resonance imaging (MRI) or computed tomography (CT) with contrast was performed to evaluate the tumor and extent of disease (Figure 1).

### 2.2 Radiotherapy and chemotherapy

All patients received radiotherapy, which included 45–50 Gy EBRT and 3–5 fractions of high‐dose rate brachytherapy (HDRB) (point A dose, 6 Gy/fraction). All IMRT cases used static beam IMRT technology. Patients with positive pelvic lymph nodes (PLNs) received a simultaneous integrated boost in the IMRT group, but they received a sequential boost in the 3DCRT group. All patients emptied the bladder and rectum 30 min before the CT scan and then drank different amount of water in 10 minutes. They were divided into two groups: the ITV group (74 patients) was determined by twice of CT scans (with a large bladder and an empty bladder at planning, with a moderate bladder at daily treatment); the N‐ITV group (403 patients) was determined by once of CT scan (with a moderate bladder at planning and daily treatment). We controlled the bladder volume by controlling the time and the amount of drinking water and monitoring by cone beam computed tomography (CBCT) at least once a week. We defined the bladder after urination, <100 cc, 100–300 cc, and >300 cc as empty, moderate, and large. The target volume delineation^23^ and constraints were determined according to Lim et al.’s consensus^10^ and the RTOG 1203 study. The PTV was expanded 5 mm in the horizontal direction and 7 mm in the craniocaudal direction from the CTV in the ITV + IMRT group and ITV + 3DCRT group. Based on the location of the tumor and the clinician's experience, the PTV was expanded by 5–10 mm in lateral, 10–20 mm in anterior, posterior, superior, and inferior directions from the CTV in the N‐ITV + IMRT group and N‐ITV + 3DCRT group. A detailed description and the schematic diagram of the ITV delineation method are shown in Table A and Figure A, separately. For bone marrow, per protocol was defined as V10 < 90% and V40 < 37%, and acceptable was V25 < 90% and V40 < 60%.

All patients received one of the following chemotherapy regimens: 1. a weekly cisplatin (DDP) regimen, 40 mg/m^2^/w DDP, w1–w5; 2. a triweekly regimen of 25 mg/m^2^/d DDP, d1–d3 + 3 g/m^2^/96 h 5‐fluorouracil (5‐FU), w1, w4; 3. a weekly TP regimen of 30 mg/m^2^ DDP + 45 mg/m^2^ paclitaxel liposome (PTXL), w1, w4; 4. a triweekly DDP + tegafur regimen of 75 mg/m^2^ DDP d1 + 0.6 g/m^2^ tegafur, d1–d3, w1, w4; 5. a weekly nedaplatin regimen of 40 mg/m^2^/w nedaplatin, w1–w5; and 6. other chemotherapy regimens: paclitaxel, capecitabine + oxaliplatin, and DDP + etoposide. Because there were very few cases, the patients with these regimens were incorporated into one group. There were 108, 36, 155, 127, 27, and 24 patients in each group, respectively (Table 1).

**TABLE 1:** Characteristics of patients and treatments (*n* = 477)

Variables	Total (%)	Radiation plan type (%)				*p* value
		N‐ITV + IMRT	ITV + IMRT	N‐ITV + 3DCRT	ITV + 3DCRT	
FIGO stage						
II	298 (62.5)	40 (52.6)	25 (61)	213 (65.1)	20 (60.6)	**0.033**
III	132 (27.7)	28 (36.8)	9 (22)	87 (26.6)	8 (24.2)	
IVA	10 (2.1)	4 (5.3)	0 (0)	6 (1.8)	0 (0)	
I	37 (7.8)	4 (5.3)	7 (17.1)	21 (6.4)	5 (15.2)	
Age (years)						
>45	410 (86)	67 (88.2)	34 (82.9)	281 (85.9)	28 (84.8)	0.885
≤45	67 (14)	9 (11.8)	7 (17.1)	46 (14.1)	5 (15.2)	
Pre‐Hb (g/L)						
<110	168 (35.2)	26 (34.2)	13 (31.7)	116 (35.5)	13 (39.4)	0.915
≥110	309 (64.8)	50 (65.8)	28 (68.3)	211 (64.5)	20 (60.6)	
Histological types						
SCC	448 (93.9)	71 (93.4)	39 (95.1)	308 (94.2)	30 (90.9)	0.873
N‐SCC	29 (6.1)	5 (6.6)	2 (4.9)	19 (5.8)	3 (9.1)	
Tumor size (cm)						
≤4	183 (38.4)	24 (31.6)	21 (51.2)	129 (39.4)	9 (27.3)	0.193
>4	259 (54.3)	44 (57.9)	17 (41.5)	175 (53.5)	23 (69.7)	
Unknown	35 (7.3)	8 (10.5)	3 (7.3)	23 (7)	1 (3)	
PLN						
Positive	143 (30)	40 (52.6)	15 (36.6)	73 (22.3)	15 (45.5)	**0.000**
Negative	334 (70)	36 (47.4)	26 (63.4)	254 (77.7)	18 (54.5)	
EBRT technique						
IMRT	117 (24.5)	76 (100)	41 (100)	0 (0)	0 (0)	**0.000**
3DCRT	360 (75.5)	0 (0)	0 (0)	327 (100)	33 (100)	
Tumor delineation method						
ITV	74 (15.5)	0 (0)	41 (100)	0 (0)	33 (100)	**0.000**
N‐ITV	403 (84.5)	76 (100)	0 (0)	327 (100)	0 (0)	
Chemotherapy regimens						
DDP + 5FU	36 (7.5)	7 (9.2)	5 (12.2)	24 (7.3)	0 (0)	**0.000**
DDP + Tegafur	127 (26.6)	29 (38.2)	0 (0)	98 (30)	0 (0)	
Nedaplatin	27 (5.7)	2 (2.6)	0 (0)	25 (7.6)	0 (0)	
Others	24 (5)	10 (13.2)	4 (9.8)	9 (2.8)	1 (3)	
DDP + PTX	155 (32.5)	17 (22.4)	3 (7.3)	134 (41)	1 (3)	
DDP	108 (22.6)	11 (14.5)	29 (70.7)	37 (11.3)	31 (93.9)	
Chemotherapy cycles (%)						
>80^a^	393 (82.4)	70 (92.1)	33 (80.5)	262 (80.1)	28 (84.8)	0.096
≤80	84 (17.6)	6 (7.9)	8 (19.5)	65 (19.9)	5 (15.2)	
Residual tumor						
Yes	204 (42.8)	23 (30.3)	24 (58.5)	141 (43.1)	16 (48.5)	**0.024**
No	273 (57.2)	53 (69.7)	17 (41.5)	186 (56.9)	17 (51.5)	
HDRB (fractions)						
≤4	310 (65)	38 (50)	27 (65.9)	226 (69.1)	19 (57.6)	**0.013**
>4	167 (35)	38 (50)	14 (34.1)	101 (30.9)	14 (42.4)	
EQD2 (Gy)						
>85	136 (28.5)	34 (44.7)	2 (4.9)	90 (27.5)	10 (30.3)	**0.000**
≤85	341 (71.5)	42 (55.3)	39 (95.1)	237 (72.5)	23 (69.7)	
Treatment time (days)						
>56	218 (45.7)	61 (80.3)	14 (34.1)	131 (40.1)	12 (36.4)	**0.000**
≤56	259 (54.3)	15 (19.7)	27 (65.9)	196 (59.9)	21 (63.6)	
Age (years), median (range)	54 (26, 79)	54 (34–75)	55 (36–70)	54 (26–79)	55 (39–70)	0.972
Pre‐Hb (g/L), median (range)	118.5 (32.1, 158)	116 (52.7–144.8)	119 (37.2–141.2)	118.3 (32.1–158)	117.9 (61.6–141.5)	0.562
Tumor size (cm), median (range)	5 (1, 9)	5 (1–8)	4 (2–6)	4.5 (1.5–9)	5 (2.5–6)	0.179
Chemotherapy cycles, median (range)	4 (1, 8)	3 (1–8)	5 (2–6)	4 (1–8)	5 (2–5)	0.181
HDRB (fractions), median (range)	4 (1, 8)	5 (3–6)	4 (2–7)	4 (1–8)	4 (3–7)	0.279
EQD2 (Gy), median (range)	82 (54, 110)	84 (68–107)	78 (62–100)	82 (54–110)	78 (70–100)	**0.000**
Treatment time (days), median (range)	56 (31, 94)	64 (35–84)	52 (31–71)	55 (32–94)	56 (32–71)	**0.000**
Follow‐up time (PFS, months), median (range)	45 (1, 97)	42 (3–91)	46 (1–88)	46 (1–97)	43 (3–65)	0.303
Follow‐up time (OS, months), median (range)	49 (1, 119)	48 (5–91)	47 (1–88)	51 (4–119)	44 (24–65)	0.319
Total	477 (100)	76 (100)	41 (100)	327 (100)	33 (100)	

Note

Bold: Statistically significant *p* value.

Abbreviations: 3DCRT, three‐dimensional conformal radiotherapy; 5‐FU, 5‐fluorouracil; DDP, cisplatin; EBRT, external beam radiotherapy; FIGO, International Federation of Gynecology and Obstetrics; HDRB, high‐dose rate brachytherapy; IMRT, intensity‐modulated radiotherapy; ITV, internal target volume; N‐ITV, non‐internal target volume; N‐SCC, non‐squamous cell carcinoma; PLN, pelvic lymph node; Pre‐Hb, the lowest level of hemoglobin before treatment; PTXL, paclitaxel liposome; SCC, squamous cell carcinoma.

^a^ For weekly regimens >80% should receive five cycles; for triweekly regimens >80% should receive two cycles.

### 2.3 Evaluation of acute HT

The HT results are summarized according to the Common Terminology Criteria for Adverse Events version 4.0 (CTCAE V4.0). Considering the small number of patients, patients with grade 3 and 4 HT were analyzed together as the grade ≥3 HT group.

### 2.4 Statistical analysis

The Kaplan–Meier method was used to compare OS and PFS between the four groups. The log‐rank test was used to compare the differences between groups. The Cox proportional hazards model was used to screen the independent prognostic factors affecting OS and PFS. The difference in the incidence of intergroup toxicity was compared by the chi‐squared test. Binary logistic regression was screened to select the independent factors that influenced toxicity. According to the EBRT technique and tumor delineation method, all 477 cervical cancer patients were divided into four arms: Arm A, ITV + IMRT; Arm B, ITV + 3DCRT; Arm C, N‐ITV + 3DCRT; and Arm D, N‐ITV + IMRT. They became Arm A', B', C', and D', respectively, after PSM. Twenty‐four patients were excluded for undergoing other chemotherapy regimens. The remaining 453 patients were divided into two chemotherapy groups: one group (144 patients) received DDP or DDP + 5‐FU, which the NCCN guidelines strongly prefer^2^ when patients receive CCRT, while the other patients were regarded as one group (309 cases). We implemented PSM in SPSS 24.0 to pair Arm C with Arm D, Arm A with Arm D', and Arm B with Arm C'. The PSM method was carried out at a ratio of 1:1 to match the four arms. FIGO stage, tumor size (cm), chemotherapy regimen, and pelvic lymph node (PLN) status were matched as covariates. The choice of matching covariates is based on the results of toxicity analysis in our study. Match tolerance was set up appropriately to ensure a large enough sample size. All patients' (92 cases) plans after PSM were designed to receive 45 Gy of EBRT. Differences in characteristics and toxicity rates were detected by the *t*‐test, the chi‐squared test, or the Fisher's exact test between the four arms. The dose–volume parameters were analyzed by the Kruskal–Wallis test. *p* < 0.05 was considered significant.

## 3 RESULTS

The median follow‐up times were 49 months (1–119) and 45 months (1–97) for OS and PFS, respectively. The follow‐up rates for OS and PFS were 89.5% and 91.4%, respectively. Three (4.1%) patients in the ITV group and 38 (9.4%) in the N‐ITV group were completely lost to follow‐up. The 4‐year OS rates were 77.9%, 71.5%, 61.4%, and 29.6% for stage I, II, III, and IVA patients, respectively (Table 2). The 2‐year, 3‐year, and 5‐year OS and PFS rates were 80.1%, 74.0%, and 64.1% and 72.9%, 66.4%, and 58.3%, respectively.

**TABLE 2:** Results of univariate and multivariate Cox proportional hazards model for OS (*n* = 427) and PFS (*n* = 436)

Variables	OS						PFS					
	Univariate				Multivariate		Univariate				Multivariate	
	*n* (%)	4‐year OS	HR (96% CI)	*p* value	HR (95% CI)	*p* value	*n* (%)	4‐year PFS	HR (96% CI)	*p* value	HR (96% CI)	*p* value
FIGO stage												
I	34 (8)	77.9	0.73 (0.35, 1.5)	0.386	0.92 (0.44, 1.92)	0.823	34 (7.8)	74.7	0.70 (0.35, 1.38)	0.301	0.90 (0.45, 1.79)	0.758
III	118 (27.6)	61.4	1.55 (1.1, 2.19)	**0.012**	1.42 (0.98, 2.06)	0.067	124 (28.4)	51.5	1.81 (1.32, 2.48)	**0.000**	1.47 (1.05, 2.07)	**0.025**
IVA	9 (2.1)	29.6	2.7 (1.18, 6.18)	**0.019**	4.11 (1.74, 9.69)	**0.001**	9 (2.1)	33.3	2.56 (1.12, 5.83)	**0.026**	3.04 (1.32, 7.03)	**0.009**
II	266 (62.3)	71.5	1		1		269 (61.7)	66.9	1		1	
Age (years)^a^												
>45	368 (86.2)	69.7	0.76 (0.49, 1.16)	0.200	1.00 (0.98, 1.02)	0.765	375 (86)	63.9	0.77 (0.51, 1.15)	0.197	0.99 (0.97, 1.01)	0.352
≤45	59 (13.8)	60.1	1				61 (14)	53.9	1			
Pre‐Hb (g/L)^a^												
≥110	278 (65.1)	70.5	0.79 (0.57, 1.10)	0.166	1.00 (0.99, 1.01)	0.938	282 (64.7)	64.9	0.75 (0.55, 1.01)	0.055	1.00 (0.99, 1.01)	0.840
<110	149 (34.9)	64.3	1				154 (35.3)	58.0	1			
Histological types												
SCC	401 (93.9)	69.6	0.53 (0.31, 0.92)	**0.024**	0.55 (0.31, 0.98)	**0.041**	410 (94)	63.8	0.58 (0.34, 0.98)	**0.042**	0.57 (0.33, 0.98)	**0.043**
N‐SCC	26 (6.1)	49.7	1		1		26 (6)	42.3	1		1	
Tumor size (cm)^a^												
>4	230 (53.9)	64.5	1.38 (0.98, 1.95)	0.065	1.07 (0.92, 1.24)	0.398	237 (54.4)	58.5	1.39 (1.01, 1.90)	**0.042**	1.01 (0.88, 1.17)	0.868
Unknown	34 (8)	79.4	0.81 (0.40, 1.64)	0.553			34 (7.8)	70.6	0.87 (0.46, 1.65)	0.661		
≤4	163 (38.2)	71.7	1				165 (37.8)	66.8	1			
PLN												
Positive	134 (31.4)	63.4	1.39 (0.99, 1.93)	0.055	1.33 (0.93, 1.89)	0.114	138 (31.7)	57.2	1.46 (1.08, 1.99)	**0.014**	1.39 (1.01, 1.92)	**0.046**
Negative	293 (68.6)	70.5	1		1		298 (68.3)	65	1		1	
EBRT technique												
IMRT	112 (26.2)	64.8	1.16 (0.82, 1.66)	0.402	1.01 (0.68, 1.52)	0.947	113 (25.9)	60.4	1.13 (0.82, 1.58)	0.456	1.02 (0.69, 1.50)	0.937
3D‐CRT	315 (73.8)	69.5	1		1		323 (74.1)	63.2	1		1	
Tumor delineation method												
ITV	71 (16.6)	75.8	0.60 (0.36, 1.01)	0.055	0.52 (0.27, 0.98)	**0.044**	71 (16.3)	72.9	0.55 (0.34, 0.89)	**0.016**	0.59 (0.36, 0.99)	**0.045**
N‐ITV	356 (83.4)	66.7	1		1		365 (83.7)	60.3	1		1	
Chemotherapy regimens												
DDP + 5FU	32 (7.5)	64.9	1.00 (0.5, 1.98)	0.991	0.62 (0.30, 1.30)	0.207	33 (7.6)	66.7	1.02 (0.53, 1.97)	0.955	0.54 (0.26, 1.10)	0.088
DDP + Tegafur	110 (25.8)	64.0	1.24 (0.79, 1.97)	0.350	0.83 (0.48, 1.43)	0.491	114 (26.1)	55.0	1.47 (0.96, 2.26)	0.078	0.94 (0.56, 1.58)	0.809
Nedaplatin	25 (5.9)	82.5	0.43 (0.15, 1.22)	0.113	0.24 (0.08, 0.73)	**0.011**	25 (5.7)	80.0	0.70 (0.31, 1.57)	0.384	0.42 (0.18, 1.02)	0.055
Others	22 (5.2)	68.2	1.30 (0.62, 2.73)	0.491	0.79 (0.33, 1.86)	0.583	22 (5)	63.6	1.54 (0.78, 3.03)	0.217	0.78 (0.35, 1.76)	0.552
DDP + PTXL	139 (32.6)	67.8	1.07 (0.69, 1.68)	0.755	0.71 (0.42, 1.21)	0.210	142 (32.6)	61.7	1.18 (0.77, 1.79)	0.455	0.80 (0.48, 1.33)	0.388
DDP	99 (23.2)	71.5	1		1		100 (22.9)	65.9	1		1	
Chemotherapy cycles (%)												
>80^b^	353 (82.7)	67.5	1.13 (0.74, 1.74)	0.571	0.92 (0.58, 1.48)	0.737	360 (82.6)	61.0	1.23 (0.82, 1.84)	0.320	1.01 (0.64, 1.58)	0.968
≤80	74 (17.3)	72.5	1		1		76 (17.4)	69.5	1		1	
Residual tumor												
Yes	183 (42.9)	62.8	1.33 (0.97, 1.83)	0.081	1.24 (0.88, 1.76)	0.222	189 (43.3)	56.8	1.38 (1.03, 1.85)	**0.031**	1.28 (0.93, 1.75)	0.124
No	244 (57.1)	72.5	1		1		247 (56.7)	66.9	1		1	
HDRB (fractions)^a^												
>4	156 (36.5)	63.6	1.49 (1.09, 2.06)	**0.014**	0.91 (0.54, 1.53)	0.724	159 (36.5)	55.9	1.55 (1.15, 2.08)	**0.004**	0.94 (0.59, 1.48)	0.776
≤4	271 (63.5)	71.2	1				277 (63.5)	66.3	1			
EQD2 (Gy)^a^												
>85	127 (29.7)	62.8	1.59 (1.15, 2.20)	**0.006**	1.03 (1.01, 1.06)	**0.021**	131 (30)	53.4	1.69 (1.25, 2.29)	**0.001**	1.03 (1.01, 1.06)	**0.005**
≤85	300 (70.3)	70.7	1				305 (70)	66.4	1			
Treatment time (days)^a^												
>56	198 (46.4)	64.8	1.29 (0.94, 1.77)	0.120	1.00 (0.98, 1.02)	0.965	204 (46.8)	58.9	1.26 (0.94, 1.69)	0.129	1.00 (0.98, 1.02)	0.909
≤56	229 (53.6)	71.2	1				232 (53.2)	65.7	1			

Note

Bold: Statistically significant *p* value.

Abbreviations: 3DCRT, three‐dimensional conformal radiotherapy; 5‐FU, 5‐fluorouracil; CI, confidence interval; DDP, cisplatin; EBRT, external beam radiotherapy; FIGO, International Federation of Gynecology and Obstetrics; HDRB, high‐dose rate brachytherapy; HR, hazard ratio; IMRT, intensity‐modulated radiotherapy; ITV, internal target volume; N‐ITV, non‐internal target volume; N‐SCC, non‐squamous cell carcinoma; OS, overall survival; PFS, progression‐free survival; PLN, pelvic lymph node; Pre‐Hb, the lowest level of hemoglobin before treatment; PTXL, paclitaxel liposome; SCC, squamous cell carcinoma.

^a^ When performing univariate analysis, continuous variables are compared by groups; when performing multivariate analysis, the original values of continuous variables are included.

^b^ For weekly regimens >80% should receive five cycles; for triweekly regimens >80% should receive two cycles.

Survival comparison of the patients with or without using the ITV delineation methods is shown in Figure B. Compared to the N‐ITV group, the ITV group had a better OS (HR (95% CI): 0.52 (0.27, 0.98), *p* = 0.044) and PFS (HR (95% CI): 0.59 (0.36, 0.99), *p* = 0.045) after multivariate analysis. There was no statistically significant difference between the IMRT and 3DCRT groups in terms of OS or PFS. Patients with >4 fractions of HDRB had a significantly worse OS (HR [95% CI]: 1.49 [1.09, 2.06], *p* = 0.014) and PFS (HR [95% CI]: 1.55 [1.15, 2.08], *p* = 0.004) than patients with ≤4 fractions of HDRB in the univariate analysis. However, HDRB fractions were not statistically significant in multivariate analysis.

Multivariate prognostic analysis showed that other factors, such as chemotherapy regimen (nedaplatin vs. DDP: HR [95% CI]: 0.24 [0.08, 0.73], *p* = 0.011), histological type (squamous cell cancer [SCC] vs. non‐squamous cell cancer [N‐SCC]: HR [95% CI]: 0.55 [0.31, 0.98], *p* = 0.041), and EQD2 (>85 Gy vs. ≤85 Gy: HR [95% CI]: 1.03 [1.01, 1.06], *p* = 0.021), were also independent prognostic factors for OS in cervical cancer patients.

Histological type (SCC vs. N‐SCC, HR [95% CI]: 0.57 [0.33, 0.98], *p* = 0.043), PLN (positive vs. negative, HR [95% CI]: 1.39 [1.01, 1.92], *p* = 0.046), and EQD2 (>85 Gy vs. ≤85 Gy: HR [95% CI]: 1.03 [1.01, 1.06], *p* = 0.005) were also independent prognostic factors for PFS in cervical cancer patients.

Of the four groups, the ITV + IMRT group had the lowest incidence of acute HT (*p* = 0.000). The N‐ITV + IMRT group had the highest incidence rates of grade 3 and grade 4 HT, at 31.6% and 14.5%, respectively (Table 3). The details of all acute toxicities are shown in Table B. Only one patient had grade 4 cystitis who was from the ITV + IMRT group. ITV + IMRT group was associated with numerically higher rates of acute severe vomiting and diarrhea when compared with the other groups. However, all the differences were not statistically significant (*p* = 0.268, 0.063, respectively).

**TABLE 3:** Acute toxicities of four radiation plan type groups (*n* = 477)

Grade	Radiation plan type (%)				Total (%)	*p* value
	N‐ITV + IMRT	ITV + IMRT	N‐ITV + 3DCRT	ITV + 3DCRT		
≥3 leukopenia	29 (38.2)	9 (22)	65 (19.9)	8 (24.2)	111 (23.3)	**0.009**
≥3 thrombocytopenia	8 (10.5)	0 (0)	9 (2.8)	1 (3)	18 (3.8)	**0.007**
≥3 neutropenia	27 (35.5)	7 (17.1)	45 (13.8)	6 (18.2)	85 (17.8)	**0.000**
Total ≥3 myelosuppression	35 (46.1)	9 (22)	74 (22.6)	8 (24.2)	126 (26.4)	**0.000**
≥3 vomiting	2 (2.6)	2 (4.9)	4 (1.2)	0 (0)	8 (1.7)	0.268
≥3 diarrhea	10 (13.2)	6 (14.6)	27 (8.3)	0 (0)	43 (9)	0.083
≥3 non‐infectious cystitis	0 (0)	1 (2.4)	0 (0)	0 (0)	1 (0.2)	**0.014**

Note

Bold: Statistically significant *p* value.

Abbreviations: 3DCRT, three‐dimensional conformal radiotherapy; HT, hematological toxicity; IMRT, intensity‐modulated radiotherapy; ITV, internal target volume; N‐ITV, non‐internal target volume.

Compared with the ITV + 3DCRT group, the N‐ITV + IMRT group had a significantly higher rate of acute radiation toxicity, and the ITV + IMRT and N‐ITV + 3DCRT groups had significantly lower rates of grade ≥3 leukopenia (38.2%, 22.0%, and 19.9% vs. 24.2%, *p* = 0.009), grade ≥3 thrombocytopenia (10.5%, 0%, and 2.8% vs. 3%, *p* = 0.007), and grade ≥3 neutropenia (35.5%, 17.1%, and 13.8% vs. 18.2%, *p* = 0.000) (Figure C). Regarding the impact of chemotherapy regimens on HT, the rates of grade ≥3 myelosuppression were lower in the DDP + PTXLs group, nedaplatin group, and DDP + tegafur group and were higher in the DDP + 5‐FU group and other groups than the DDP chemotherapy group (respectively, 16.1%, 25.9%, 27.6%, 41.7%, and 58.3% vs. 27.8%, *p* = 0.000) (Figure D).

The results of the binary logistic regression model showed that different radiation plan types (ITV + IMRT and N‐ITV + 3DCRT vs. N‐ITV + IMRT: HR [95% CI]: 0.35 [0.12, 1.01] and 0.45 [0.24, 0.82], respectively, *p* = 0.052, 0.009), different chemotherapy regimens (DDP + 5‐FU, DDP + tegafur, and others vs. DDP + PTXL: HR [95% CI]: 5.12 [2.18, 12.04], 2.05 [1.07, 3.89], and 6.66 [2.28, 19.44], *p* = 0.000, 0.029, and 0.001), and chemotherapy cycles (>80% vs. ≤80%, HR [95% CI]: 0.30 [0.16, 0.54], *p* = 0.000) might be independent risk factors for acute HT (Table 4).

**TABLE 4:** Results of univariate and multivariate binary logistic regression for grade ≥3 HT (*n* = 477)

Variables	Univariate		Multivariate	
	HR (95% CI)	*p* value	HR (95% CI)	*p* value
Tumor size (cm)^a^				
>4	0.95 (0.62, 1.47)	0.831	0.95 (0.78, 1.15)	0.577
Unknown	1.09 (0.49, 2.44)	0.827		
≤4	1	0.934		
HDRB (fractions)^a^				
≤4	0.87 (0.57, 1.33)	0.530	0.95 (0.69, 1.31)	0.743
>4	1			
PLN				
Positive	1.66 (1.08, 2.55)	**0.021**	1.06 (0.63, 1.81)	0.818
Negative	1		1	
FIGO stage				
I	0.62 (0.25, 1.54)	0.303	0.69 (0.26, 1.82)	0.452
III	1.83 (1.17, 2.85)	**0.008**	1.30 (0.79, 2.17)	0.306
Iva	0.36 (0.04, 2.85)	0.330	0.18 (0.02, 1.78)	0.144
II	1	**0.013**	1	
Radiation plan type				
ITV + 3DCRT	0.38 (0.15, 0.94)	**0.036**	0.50 (0.16, 1.56)	0.233
ITV + IMRT	0.33 (0.14, 0.78)	**0.012**	0.35 (0.12, 1.01)	0.052
N‐ITV + 3DCRT	0.34 (0.20, 0.58)	**0.000**	0.45 (0.24, 0.82)	**0.009**
N‐ITV + IMRT	1	**0.001**	1	
Chemotherapy regimens				
DDP	2.00 (1.10, 3.65)	**0.024**	1.97 (0.92, 4.21)	0.082
DDP + 5FU	3.71 (1.69, 8.17)	**0.001**	5.12 (2.18, 12.04)	**0.000**
DDP + Tegafur	1.98 (1.11, 3.53)	**0.021**	2.05 (1.07, 3.89)	**0.029**
Nedaplatin	1.82 (0.70, 4.76)	0.222	1.67 (0.60, 4.60)	0.325
Others	7.28 (2.91, 18.22)	**0.000**	6.66 (2.28, 19.44)	**0.001**
DDP + PTX	1	**0.000**	1	
Chemotherapy cycles (%)				
>80^b^	0.48 (0.29, 0.79)	**0.004**	0.30 (0.16, 0.54)	**0.000**
≤80	1		1	
EQD2 (Gy)^a^				
>85	1.59 (1.03, 2.45)	**0.038**	1.02 (0.94, 1.12)	0.606
≤85	1			
Treatment time (days)^a^				
>56	1.64 (1.09, 2.47)	**0.018**	1.02 (1.00, 1.04)	0.057
≤56	1			

Note

Bold: Statistically significant *p* value.

Abbreviations: 3DCRT, three‐dimensional conformal radiotherapy; 5‐FU, 5‐fluorouracil; CI, confidence interval; cisplatin, DDP; FIGO, International Federation of Gynecology and Obstetrics; HDRB, high‐dose rate brachytherapy; HR, hazard ratio; IMRT, intensity‐modulated radiotherapy; ITV, internal target volume; N‐ITV, non‐internal target volume; PLN, pelvic lymph node; PTXL, paclitaxel liposome.

^a^ When performing univariate analysis, continuous variables are compared by groups; when performing multivariate analysis, the original values of continuous variables are included.

^b^ For weekly regimens >80% should receive five cycles; for triweekly regimens >80% should receive two cycles.

The characteristics of the 92 patients matched by PSM are shown in Table C. The patients of the four groups had comparable characteristics. The pelvic bone marrow relative volume receiving a dose less than 35 Gy in the N‐ITV + IMRT group was greater than those in the ITV + IMRT group and the N‐ITV + 3DCRT group (*p* < 0.05) (Table 5).

**TABLE 5:** Dose–volume histogram comparisons for PTV and main OARs in different radiation plan types (*n* = 92)

	N‐ITV + 3DCRT vs. ITV + IMRT	N‐ITV + 3DCRT vs. ITV + 3DCRT	N‐ITV + 3DCRT vs. N‐ITV + IMRT	ITV + IMRT vs. ITV + 3DCRT	ITV + IMRT vs. N‐ITV + IMRT	ITV + 3DCRT vs. N‐ITV + IMRT
PTV (cc)	1766.11 vs. 1241.65*	1766.11 vs. 1248.84	1766.11 vs. 1517.88	1241.65 vs. 1248.84	1241.65 vs. 1517.88**	1248.84 vs. 1517.88*
Bone marrow						
V10 (%)	89.98 vs. 92.10	89.98 vs. 94.62	89.98 vs. 97.16**	92.1 vs. 94.62	92.1 vs. 97.16**	94.62 vs. 97.16*
V20 (%)	82.62 vs. 81.62	82.62 vs. 89.95**	82.62 vs. 89.71**	81.62 vs. 89.95*	81.62 vs. 89.71*	89.95 vs. 89.71
V30 (%)	58.49 vs. 59.33	58.49 vs. 64.75	58.49 vs. 67.79*	59.33 vs. 64.75	59.33 vs. 67.79*	64.75 vs. 67.79
V40 (%)	40.90 vs. 30.20*	40.90 vs. 41.18**	40.90 vs. 37.49**	30.20 vs. 41.18	30.20 vs. 37.49	41.18 vs. 37.49
V45 (%)	30.98 vs. 16.83*	30.98 vs. 33.03**	30.98 vs. 25.82**	16.83 vs. 33.03	16.83 vs. 25.82	33.03 vs. 25.82
Dmean (Gy)	32.12 vs. 31.48	32.12 vs. 34.39*	32.12 vs. 33.05	31.48 vs. 34.39**	31.48 vs. 33.05	34.39 vs. 33.05
Bladder						
V10 (%)	99.98 vs. 99.99	99.98 vs. 99.99	99.98 vs. 99.99	99.99 vs. 99.99	99.99 vs. 99.99	99.99 vs. 99.99
V20 (%)	99.98 vs. 98.69	99.98 vs. 99.99	99.98 vs. 99.39	98.69 vs. 99.99	98.69 vs. 99.39	99.99 vs. 99.39
V30 (%)	95.13 vs. 87.29*	95.13 vs. 98.61	95.13 vs. 90.80	87.29 vs. 98.61**	87.29 vs. 90.80	98.61 vs. 90.80**
V40 (%)	79.58 vs. 58.91**	79.58 vs. 83.10	79.58 vs. 69.61	58.91 vs. 83.10**	58.91 vs. 69.61	83.10 vs. 69.61*
V45 (%)	60.15 vs. 38.14*	60.15 vs. 73.48	60.15 vs. 46.76	38.14 vs. 73.48**	38.14 vs. 46.76	73.48 vs. 46.76**
Dmean (Gy)	43.81 vs. 40.11**	43.81 vs. 44.59	43.81 vs. 41.78	40.11 vs. 44.59**	40.11 vs. 41.78	44.59 vs. 41.78*
Volume (cc)	139.73 vs. 393.72**	139.73 vs. 352.56**	139.73 vs. 199.94	393.72 vs. 352.56	393.72 vs. 199.94**	352.56 vs. 199.94*
Rectum						
V10 (%)	99.48 vs. 99.96	99.48 vs. 99.98	99.48 vs. 99.97	99.96 vs. 99.98	99.96 vs. 99.97	99.98 vs. 99.97
V20 (%)	99.13 vs. 99.87	99.13 vs. 99.98	99.13 vs. 99.94	99.87 vs. 99.98	99.87 vs. 99.94	99.98 vs. 99.94
V30 (%)	97.56 vs. 97.60	97.56 vs. 99.98**	97.56 vs. 98.73	97.60 vs. 99.98**	97.60 vs. 98.73	99.98 vs. 98.73
V40 (%)	85.37 vs. 86.86	85.37 vs. 95.39*	85.37 vs. 89.65	86.86 vs. 95.39	86.86 vs. 89.65	95.39 vs. 89.65
V45 (%)	47.26 vs. 60.45	47.26 vs. 81.75*	47.26 vs. 47.17	60.45 vs. 81.75*	60.45 vs. 47.17	81.75 vs. 47.17**
Dmean (Gy)	43.35 vs. 44.02	43.35 vs. 45.65*	43.35 vs. 44.14	44.02 vs. 45.65	44.02 vs. 44.14	45.65 vs. 44.14
Small bowel sac						
V10 (%)	83.53 vs. 80.55	83.53 vs. 77.67	83.53 vs. 78.64	80.55 vs. 77.67	80.55 vs. 78.64	77.67 vs. 78.64
V20 (%)	73.59 vs. 65.50	73.59 vs. 67.21	73.59 vs. 66.36	65.50 vs. 67.21	65.50 vs. 66.36	67.21 vs. 66.36
V30 (%)	54.24 vs. 36.19**	54.24 vs. 39.62*	54.24 vs. 39.47**	36.19 vs. 39.62	36.19 vs. 39.47	39.62 vs. 39.47
V40 (%)	34.22 vs. 17.62**	34.22 vs. 21.91*	34.22 vs. 21.38*	17.62 vs. 21.91	17.62 vs. 21.38	21.91 vs. 21.38
V45 (%)	25.93 vs. 10.79**	25.93 vs. 16.50	25.93 vs. 13.21**	10.79 vs. 16.50	10.79 vs. 13.21	16.50 vs. 13.21
Dmean (Gy)	30.00 vs. 25.08*	30.00 vs. 25.85*	30.00 vs. 25.53*	25.08 vs. 25.85	25.08 vs. 25.53	25.85 vs. 25.53
V45 (cc)	242.15 vs. 182.27**	242.15 vs. 146.51*	242.15 vs. 145.26**	182.27 vs. 146.51	182.27 vs. 145.26	146.51 vs. 145.26
Bowel sac						
V10 (%)	76.55 vs. 74.88	76.55 vs. 74.78	76.55 vs. 75.04	74.88 vs. 74.78	74.88 vs. 75.04	74.78 vs. 75.04
V20 (%)	66.66 vs. 58.73	66.66 vs. 64.57	66.66 vs. 61.86	58.73 vs. 64.57	58.73 vs. 61.86	64.57 vs. 61.86
V30 (%)	47.03 vs. 32.77**	47.03 vs. 35.95*	47.03 vs. 36.64*	32.77 vs. 35.95	32.77 vs. 36.64	35.95 vs. 36.64
V40 (%)	31.39 vs. 17.17**	31.39 vs. 21.11*	31.39 vs. 20.79*	17.17 vs. 21.11	17.17 vs. 20.79	21.11 vs. 20.79
V45 (%)	24.87 vs. 11.00**	24.87 vs. 16.61*	24.87 vs. 13.70**	11.00 vs. 16.61	11.00 vs. 13.70	16.61 vs. 13.70
Dmean (Gy)	27.54 vs. 23.37*	27.54 vs. 24.83	27.54 vs. 24.38	23.37 vs. 24.83	23.37 vs. 24.38	24.83 vs. 24.38

Note

* *p* < 0.05; **p < 0.001.

Abbreviations: 3DCRT, three‐dimensional conformal radiotherapy; Dmean, mean dose; IMRT, intensity‐modulated radiotherapy; ITV, internal target volume; N‐ITV, non‐internal target volume; PTV, planning target volume (cc); Vx, volume (%) receiving at least x Gy.

## 4 DISCUSSION

This is the first study comparing survival rates between ITV and N‐ITV groups by controlling the bladder volume and considering different delineation method in cervical cancer patients receiving CCRT. This is the first study to analyze the difference between four groups (N‐ITV + IMRT, ITV + IMRT, N‐ITV + 3DCRT, and ITV + 3DCRT) in the treatment of cervical cancer.

Like many studies,^4^, ^24^, ^25^, ^26^, ^27^, ^28^ our study discovered that IMRT for CCRT of cervical cancer did not worsen the survival outcomes compared to 3DCRT. Our study showed that the 2‐year, 3‐year, and 5‐year OS rates in IMRT and 3DCRT groups were 76.8%, 75.0%, and 58.3% and 81.3%, 73.7%, and 66.1%, respectively. These survival rates were similar even higher to the long‐term follow‐up results of a multi‐institutional phase 2 study^29^ by Kato et al., in which the 5‐year OS rate was 55.1%. There was no statistically significant difference in survival analysis between IMRT and 3DCRT groups in our study, which is the same as several prospective randomized studies^24^, ^26^, ^29^ or meta‐analysis.^25^

Based on the concept of ITV proposed in ICRU62,^30^ a large amount of studies^11^, ^12^, ^15^, ^17^, ^18^, ^19^, ^20^, ^21^, ^22^ mainly concentrated on organ motion or contouring margins using the ITV delineation method. However, to our knowledge, there is no study comparing the prognostic difference between the ITV group and the N‐ITV group in single radiotherapy techniques such as IMRT and 3DCRT. After considering, but not limited to the effects of different radiation techniques, our study showed that the implementation of ITV was an independent favorable prognostic factor for OS and PFS. It is worthy to mention that in one recent study^18^ by Niyoteka et al, they found that it was not enough to ensure adequate dose coverage in the high‐risk CTV even though using the ITV concept. There is no doubt that not only the ITV delineation method, but the bladder volume also played an important role in the treatment of cervical cancer. CBCT motoring and online adaptive radiotherapy^19^, ^31^, ^32^, ^33^, ^34^, ^35^ are recommended among in cervical cancer.

Although several studies showed IMRT was useful to decrease the gastrointestinal toxicity^4^, ^5^, ^24^, ^25^, ^26^, ^36^ and genitourinary toxicity,^5^, ^25^ our study showed that the different incidence of gastrointestinal toxicity in IMRT and 3DCRT groups was not statistically significant. Only two patients in ITV group had grade ≥3 non‐infectious cystitis. Acute gastrointestinal toxicity and genitourinary system toxicity in this study were based on patient medical records and nursing records. And we did not collect late toxicity.

In terms of acute HT between IMRT and 3DCRT, different studies had different results. A randomized prospective study by Naik et al.^37^ and a national multicenter study by Erpolat et al.^38^ both showed that there was no significant difference in HT between IMRT and 3DCRT. Mell et al considered that IMRT reduced acute HT compared to 3DCRT. The incidence of grade ≥3 HT in the Mell et al's study^39^ was 38.6%. According to laboratory test results, our study showed that the incidence of acute grade ≥3 HT in IMRT and 3DCRT groups was 37.6% and 22.8%, respectively. It was similar even lower than several studies.^5^, ^39^, ^40^ However, our study showed that the incidence of acute grade ≥3 HT in N‐ITV + IMRT group (46.2%) was higher than ITV + IMRT group (22.0%) and N‐ITV + 3DCRT group (22.6%).

The study^41^ by Albuquerque et al. discovered the correlation between bone marrow volume and HT, and the study^42^ by Brixey et al. discovered that whole pelvic radiation had an impact on acute HT. Therefore, we performed a dose–volume analysis after PSM. Considering the bladder volume could also have an effect on dose–volume parameters of organs at risk, we compared the bladder volume at planning. The difference in bladder volumes in N‐ITV + IMRT (199.92 cc) and N‐ITV + 3DCRT (139.73 cc) groups was not statistically significant (*p* ≥ 0.05). But a low‐dose relative volume (V10, V20, and V30) of pelvic bone marrow in N‐ITV + IMRT group was higher than N‐ITV + 3DCRT group (*p* < 0.05). As shown in Rose et al's research^43^ and Chang et al's research,^40^ the low‐dose relative volume of pelvic bone marrow was valuable in predicting HT. In addition, the combination of the implication of ITV and the large bladder suggested a lower low‐dose volume of pelvic bone marrow (ITV + IMRT vs. N‐ITV + IMRT, *p* < 0.05).

Among previous studies,^4^, ^44^ IMRT decreased high‐dose volume of organ at risk. Our study also suggested that the IMRT was associated with decreasing the high‐dose relative volume (V30, V40, and V45) of the small bowel apace and the bowel space (N‐ITV + IMRT vs. N‐ITV + 3DCRT, *p* < 0.05), and IMRT was associated with decreasing the high‐dose relative volume (V30, V40, and V45) of the bladder (ITV + IMRT vs. ITV + 3DCRT, *p* < 0.05). These bowel and bladderdose–volume differences were not translated in gastrointestinal toxicity and genitourinary toxicity in this study. Further study is needed to discover the potential impact factor.

Regarding chemotherapy, nedaplatin was an independent prognostic factor for OS. However, Li et al. did not support the use of nedaplatin in place of DDP in the treatment of patients who received CCRT.^45^ DDP + 5FU and DDP + tegafur regimens had a higher incidence of HT than DDP + PTX in our study. Patients who experienced HT during chemotherapy were likely to receive less chemotherapy, which might explain why a higher chemotherapy completion rate was associated with a lower incidence of HT. Prospective research is also needed to further evaluate the impacts of different chemotherapy approaches.

Patients who received >4 HDRB fractions had worse OS and PFS in univariate analysis. Patients with residual tumors after four fractions of HDRB had poorer prognoses. This could be explained by the following three reasons. First, their tumor volume was large before treatment. Second, the tumor itself was relatively less sensitive to radiation therapy and chemotherapy. Third, >4 fractions of HDRB might be associated with a longer overall treatment time. Survival might also have been influenced by other factors. In multivariate analysis, HDRB was not an independent influencing factor for survival.

It does have the limitations of a retrospective study, including variability in the patient population and the differing sizes of the four treatment groups evaluated. Like any retrospective study, selection bias may be unavoidable. Another limitation is that we cannot neglect the difference in bladder volume to discuss the role of ITV in our research. In Yaparpal et al's research,^46^ they thought that full bladder planning was not necessary. Eminowi et al.^47^ directly recommended bladder volumes of 150–300 cc at planning, which were similar to that of N‐ITV group in our study. Whatever, the monitoring and control of bladder volume are worthy to be studied at planning and daily treatment. Further research is needed to evaluate the clinical significance of IMRT techniques and ITV delineation methods.

## 5 CONCLUSIONS

The implementation of the ITV delineation method was an independent favorable prognostic factor for OS and PFS in cervical cancer patients receiving CCRT. The ITV delineation method should be used in combination with IMRT to decrease the pelvic bone marrow relative V5–30 and thus decrease the incidence of grade ≥3 HT. Without ITV, IMRT may be a worse choice than 3DCRT in the treatment of cervical cancer.

## REVIEW BOARD/COMMITTEE APPROVAL

This study was approved by the Regional Ethics Committee of our hospital.

## CONFLICT OF INTEREST

No potential conflict of interest relevant to this article was reported.

## AUTHOR CONTRIBUTION

Liang‐Xi Xie, Tian‐Tian Zhai, and Elizabeth A. Kidd designed and supervised the study. Yu‐Qin Liang, Sen‐Quan Feng, Wen‐Jia Xie, and Qiong‐Zhi Jiang acquired the data. Yu‐Qin Liang, Yan‐Fen Yang, and Ren Luo conducted the analyses. Yu‐Qin Liang wrote the full draft of the article. All authors validated the analysis and study results and revised the article critically. All authors were responsible for the interpretation of data and writing the report. All authors have final approval of the version to be submitted.

## Supporting information

Figure S1

Figure S2

Figure S3

Figure S4

Tables S1–S3

## Data Availability

Research data are stored in an institutional repository and will be shared upon request to the corresponding author.
