# Supplementary figures and images for: Comparison of survival, acute toxicities, and dose–volume parameters between intensity‐modulated radiotherapy with or without internal target volume delineation method and three‐dimensional conformal radiotherapy in cervical cancer patients: A retrospective and propensity score‐matched analysis
Source: Cancer Med. 2021 Nov 24;11(1):151–65. doi: 10.1002/cam4.4439 (PMC8704157; doi:10.1002/cam4.4439)

- Bladder
- Bowel sac
- CTV
- CTV\_e
- CTV\_f
- CTVn
- GTVn
- GTVp
- ITV
- Pelvic bones
- PTV
- Rectum
- Small bowel sac

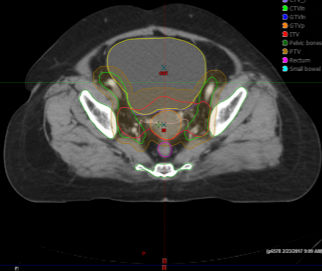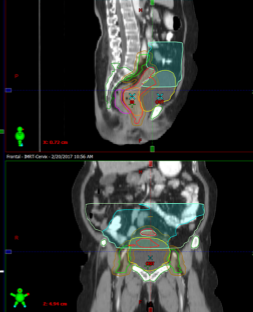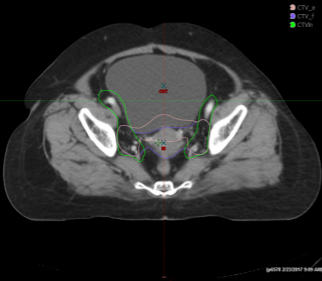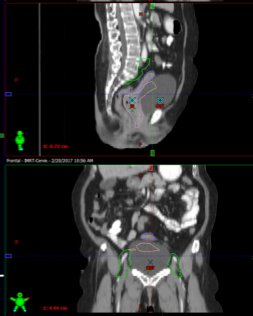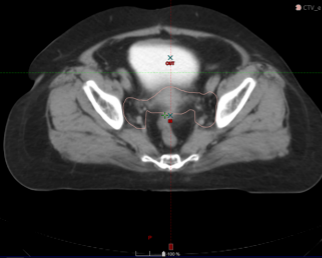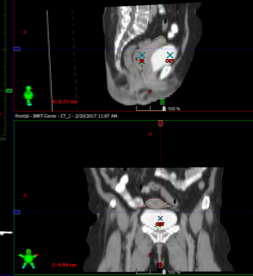

Supplement: Supplementary file 1 — Figure S1 [file CAM4-11-151-s003.pdf]

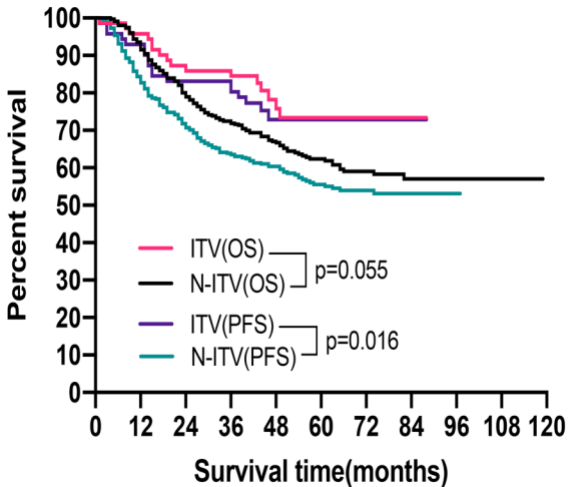

Supplement: Supplementary file 2 — Figure S2 [file CAM4-11-151-s004.pdf]

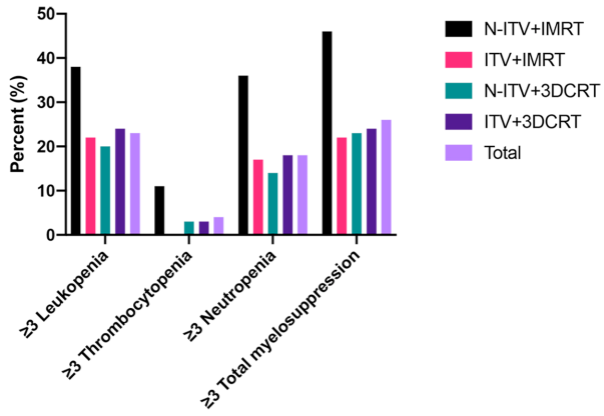

Supplement: Supplementary file 3 — Figure S3 [file CAM4-11-151-s005.pdf]

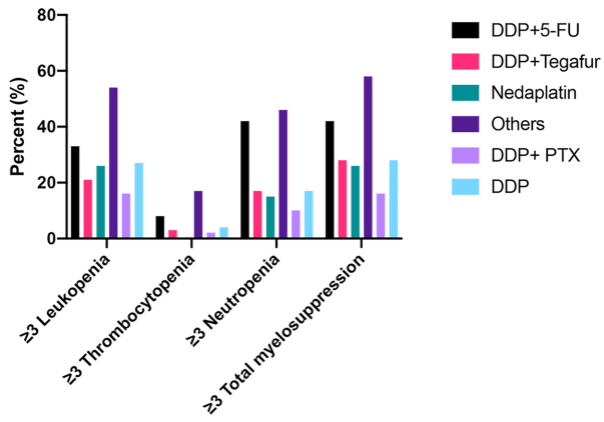

Supplement: Supplementary file 4 — Figure S4 [file CAM4-11-151-s002.pdf]
