# Supplementary material for: Comparison of survival, acute toxicities, and dose–volume parameters between intensity‐modulated radiotherapy with or without internal target volume delineation method and three‐dimensional conformal radiotherapy in cervical cancer patients: A retrospective and propensity score‐matched analysis
Source: Cancer Med. 2021 Nov 24;11(1):151–65. doi: 10.1002/cam4.4439 (PMC8704157; doi:10.1002/cam4.4439)
Supplement: Supplementary file 5 — Tables S1–S3 [file CAM4-11-151-s001.docx]

| **Supplementary table/figure legends**  Table A. Details of the target volume delineation  *Abbreviations:* CBCT, Cone Beam Computed Tomography; GTVp, Gross Target Volume of Primary; GTVln, Gross Target Volume of lymph nodes; CTV, Clinical Target volume; PTV, Planning Target Volume; ITV, Internal Target Volume; N-ITV, Non-Internal Target Volume; OARs, Organs at Risk.  Table B. Details of all acute toxicities (n=477).  *Abbreviations:* IMRT, Intensity Modulated Radiotherapy; 3DCRT, 3-dimensional Conformal Radiotherapy; ITV, Internal Target Volume; N-ITV, Non-Internal Target Volume.  Table C. Characteristics of patients and treatments (n=92).  ^†^ Guidelines prefer: Cisplatin or Cisplatin +5-fluorouracil;  *Abbreviations:* FIGO, International Federation of Gynecology and Obstetrics; Cisplatin, DDP; PTXL, Paclitaxel Liposome; HDRB, High-Dose-Rate Brachytherapy; PLN, Pelvic Lymph Node; ITV, Internal Target Volume; N-ITV, Non-Internal Target Volume; IMRT, Intensity-Modulated Radiotherapy; 3DCRT, 3-dimensional Conformal Radiotherapy; HR, Hazard Ratio; CI, Confidence Interval.  Bold: Statistically significant P-value.  Fig. A. The schematic diagram of the ITV delineation method.  *Abbreviations:* ITV, Internal Target Volume.  Fig. B. Survival comparison of the 2 tumor delineation methods.  *Abbreviations:* ITV, Internal Target Volume; N-ITV, Non-Internal Target Volume; OS, Overall Survival; PFS, Progression-free Survival.  Fig. C. Acute HT comparison of the 4 radiation plan types.  *Abbreviations:* IMRT, Intensity-Modulated Radiotherapy; 3DCRT, 3-dimensional Conformal Radiotherapy; ITV, Internal Target Volume; N-ITV, Non-Internal Target Volume.  Fig. D. Acute HT comparison of 6 chemotherapy regimens.  *Abbreviations:* DDP; 5-FU, 5-fluorouracil; PTXL, Paclitaxel Liposome; HT, Hematological Toxicity.  Table A. Details of the target volume delineation | | |
| --- | --- | --- |
|  | ITV | N-ITV |
| CT scan at planning | Twice (Large bladder over 300cc, and empty bladder less than 100cc after urination) | Once (100-300cc moderate bladder) |
| CBCT at treatment | 100-300cc moderate bladder | 100-300cc moderate bladder (same preparation as planning) |
| GTVp | Visible tumor mass, high signal on T2-weighted MRI images. | Same |
| GTVln | Visible or suspicious lymph nodes. | Same |
| CTV_f | Delineated on large bladder status image. Includes GTVp, uterus, parametria, ovaries, and 30mm vaginal tissue blow cervix or GTVp. | Not exist |
| CTV_e | Delineated on empty bladder status image. Includes GTVp, uterus, parametria, ovaries, and 30mm vaginal tissue blow cervix or GTVp. | Not exist |
| ITV | The merge of CTV-f and CTV-e. | Not exist |
| CTVln | Visible or suspicious lymph nodes, together with draining nodal region, (common, internal, and external iliac lymph node regions, and obturator and presacral lymph node regions. Abdominal aortic lymph node region will be included if para-aortic lymph node enlargement. 7mm margin surrounding blood vessels, 15mm anterior to the vertebra and sacrum. Bones, muscle and bladder are excluded. Common iliac lymph nodes region: From the level of bifurcation of common iliac arteries into external and internal iliac arteries. External iliac lymph nodes Internal iliac lymph nodes region: From level of bifurcation of common iliac artery into external artery to level of superior aspect of femoral head where it becomes femoral artery. Internal iliac lymph nodes region: From level of bifurcation of common iliac artery into internal iliac artery, along its branches (obturator, hypogastric) terminating in paravaginal tissues at level of vaginal cuff. | Included in CTV |
| CTV | The merge of ITV and CTVln. | Includes GTVp, uterus, parametria, ovaries. And visible or suspicious lymph nodes, together with draining nodal region, (common, internal, and external iliac lymph node regions, and obturator and presacral lymph node draining regions. Abdominal aortic lymph node region will be included if para-aortic lymph node enlargement. 7mm margin surrounding blood vessels, 15mm anterior to the vertebra and sacrum. Bones, muscle and bladder are excluded.  Common iliac lymph nodes region: From the level of bifurcation of common iliac arteries into external and internal iliac arteries. External iliac lymph nodes Internal iliac lymph nodes region: From level of bifurcation of common iliac artery into external artery to level of superior aspect of femoral head where it becomes femoral artery. Internal iliac lymph nodes region: From level of bifurcation of common iliac artery into internal artery, along its branches (obturator, hypogastric) terminating in paravaginal tissues at level of vaginal cuff.  The whole uterus and parametria, exclude tissue posterior to uterosacral ligament if rectum is not invaded. Vaginal cuff and 30mm of vagina inferior to GTVp. |
| PTV | Extended from CTV by 5mm horizontally, 7mm cranially and caudally. | Expanded by 5-10mm in lateral, 10-20mm in anterior, posterior, superior and inferior directions from CTV |
| OARs | bladder, rectum, pelvic bones (including sacrum, pubic and ischium), small bowel sac range from 10mm above the first cranial plane of CTV. | Same |

*Abbreviations:* CBCT, Cone Beam Computed Tomography; GTVp, Gross Target Volume of Primary; GTVln, Gross Target Volume of lymph nodes; CTV, Clinical Target volume; PTV, Planning Target Volume; ITV, Internal Target Volume; N-ITV, Non-Internal Target Volume; OARs, Organs at Risk.

Table B. Details of all acute toxicities (n=477).

|  |  | Radiation plan type (%) | | | | Total |
| --- | --- | --- | --- | --- | --- | --- |
|  |  | N-ITV+IMRT | ITV+IMRT | N-ITV+3DCRT | ITV+3DCRT |  |
| Leukopenia | Grade 1 | 9(11.8) | 9(22) | 85(26) | 10(30.3) | 113(23.7) |
|  | Grade 2 | 37(48.7) | 22(53.7) | 152(46.5) | 12(36.4) | 223(46.8) |
|  | Grade 3 | 22(28.9) | 7(17.1) | 60(18.3) | 8(24.2) | 97(20.3) |
|  | Grade 4 | 7(9.2) | 2(4.9) | 5(1.5) | 0(0) | 14(2.9) |
| Thrombocytopenia | Grade 1 | 24(31.6) | 4(9.8) | 34(10.4) | 4(12.1) | 66(13.8) |
|  | Grade 2 | 10(13.2) | 3(7.3) | 19(5.8) | 0(0) | 32(6.7) |
|  | Grade 3 | 6(7.9) | 0(0) | 2(0.6) | 1(3) | 9(1.9) |
|  | Grade 4 | 2(2.6) | 0(0) | 7(2.1) | 0(0) | 9(1.9) |
| Neutropenia | Grade 1 | 10(13.2) | 11(26.8) | 86(26.3) | 10(30.3) | 117(24.5) |
|  | Grade 2 | 28(36.8) | 8(19.5) | 95(29.1) | 6(18.2) | 137(28.7) |
|  | Grade 3 | 18(23.7) | 5(12.2) | 37(11.3) | 5(15.2) | 65(13.6) |
|  | Grade 4 | 9(11.8) | 2(4.9) | 8(2.4) | 1(3) | 20(4.2) |
| Myelosuppression | Grade 1 | 9(11.8) | 9(22) | 84(25.7) | 11(33.3) | 113(23.7) |
|  | Grade 2 | 31(40.8) | 22(53.7) | 145(44.3) | 12(36.4) | 210(44) |
|  | Grade 3 | 24(31.6) | 7(17.1) | 63(19.3) | 7(21.2) | 101(21.2) |
|  | Grade 4 | 11(14.5) | 2(4.9) | 11(3.4) | 1(3) | 25(5.2) |
| Vomiting | Grade 1 | 20(26.3) | 10(24.4) | 66(20.2) | 5(15.2) | 101(21.2) |
|  | Grade 2 | 24(31.6) | 7(17.1) | 74(22.6) | 3(9.1) | 108(22.6) |
|  | Grade 3 | 2(2.6) | 2(4.9) | 4(1.2) | 0(0) | 8(1.7) |
| Diarrhea | Grade 1 | 14(18.4) | 3(7.3) | 84(25.7) | 8(24.2) | 109(22.9) |
|  | Grade 2 | 26(34.2) | 12(29.3) | 91(27.8) | 15(45.5) | 144(30.2) |
|  | Grade 3 | 10(13.2) | 6(14.6) | 27(8.3) | 0(0) | 43(9) |
| Cystitis noninfective | Grade 1 | 17(22.4) | 4(9.8) | 37(11.3) | 2(6.1) | 60(12.6) |
|  | Grade 2 | 2(2.6) | 0(0) | 3(0.9) | 0(0) | 5(1) |
|  | Grade 3 | 0(0) | 1(2.4) | 0(0) | 0(0) | 1(0.2) |

*Abbreviations:* IMRT, Intensity Modulated Radiotherapy; 3DCRT, 3-dimensional Conformal Radiotherapy; ITV, Internal Target Volume; N-ITV, Non-Internal Target Volume.

| Table C. Characteristics of patients and treatments (n=92). | | | | | | | |
| --- | --- | --- | --- | --- | --- | --- | --- |
| Variables |  | Radiation plan type (%) | | | | | P-value |
|  |  | N-ITV+IMRT | ITV+IMRT | N-ITV+3DCRT | ITV+3DCRT | Total (%) |  |
| FIGO stage | I+II | 16 (23.9) | 18 (26.9) | 15 (22.4) | 18 (26.9) | 67 (100) | 0.686 |
|  | III+IVA | 7 (28) | 5 (20) | 8 (32) | 5 (20) | 25 (100) |  |
| Tumor size (cm) | ≤4 | 8 (21.1) | 13 (34.2) | 10 (26.3) | 7 (18.4) | 38 (100) | 0.270 |
|  | >4 | 13 (27.7) | 9 (19.1) | 10 (21.3) | 15 (31.9) | 47 (100) |  |
| Chemotherapy regimens | Guidelines preferred^†^ | 21 (24.4) | 22 (25.6) | 21 (24.4) | 22 (25.6) | 86 (100) | 0.870 |
|  | DDP+ PTXL | 2 (33.3) | 1 (16.7) | 2 (33.3) | 1 (16.7) | 6 (100) |  |
| Residual Tumor | Yes | 6 (17.1) | 13 (37.1) | 6 (17.1) | 10 (28.6) | 35 (100) | 0.093 |
|  | No | 17 (29.8) | 10 (17.5) | 17 (29.8) | 13 (22.8) | 57 (100) |  |
| HDRB (fractions) | ≤4 | 10 (18.9) | 16 (30.2) | 13 (24.5) | 14 (26.4) | 53 (100) | 0.342 |
|  | >4 | 13 (33.3) | 7 (17.9) | 10 (25.6) | 9 (23.1) | 39 (100) |  |
| PLN | Positive | 10 (23.8) | 10 (23.8) | 12 (28.6) | 10 (23.8) | 42 (100) | 0.913 |
|  | Negative | 13 (26) | 13 (26) | 11 (22) | 13 (26) | 50 (100) |  |

^†^ Guidelines preferred: Cisplatin or Cisplatin +5-fluorouracil;

*Abbreviations:* FIGO, International Federation of Gynecology and Obstetrics; Cisplatin, DDP; PTXL, Paclitaxel Liposome; HDRB, High Dose Rate Brachytherapy; PLN, Pelvic Lymph Node; ITV, Internal Target Volume; N-ITV, Non-Internal Target Volume; IMRT, Intensity Modulated Radiotherapy; 3DCRT, 3-dimensional Conformal Radiotherapy; HR, Hazard Ratio; CI, Confidence Interval.

Bold: Statistically significant P-value.
